# Perspectives on “Disease” and “Disability” in Child Health: The Case of Childhood Neurodisability

**DOI:** 10.3389/fpubh.2016.00226

**Published:** 2016-10-26

**Authors:** Anton Rodney Miller, Peter Rosenbaum

**Affiliations:** ^1^Department of Pediatrics, University of British Columbia, Vancouver, BC, Canada; ^2^BC Children’s Hospital Research Institute, Vancouver, BC, Canada; ^3^Department of Pediatrics, McMaster University, Hamilton, ON, Canada; ^4^CanChild Centre for Childhood Disability Research, Hamilton, ON, Canada

**Keywords:** childhood neurodisability, International Classification of Functioning, Disability and Health, human functioning, rehabilitation, biomedical diagnostic–therapeutics, nomothetic and idiographic knowledge

## Abstract

Chronic health conditions are often associated with what is termed disability. Traditional thinking has focused on diagnosis and treatment of chronic diseases and disorders, with less attention to people’s functional abilities and their contextual determinants. Understanding all of these factors is integral to addressing the predicaments and needs of persons with chronic conditions. However, these complementary yet distinct “worldviews” reflected in what we call disease and disability perspectives often remain, at best, only vaguely articulated. In this paper, we explore and expand on these perspectives in light of conceptual advances, specifically the framework of the World Health Organization’s International Classification of Functioning, Disability and Health, and their epistemic underpinnings with reference to Wilhelm Windelband’s notions of nomothetic and idiographic types of knowledge. Our primary focus is the children with neurodisability – life-long conditions that onset early in life and have functional consequences that impact developmental trajectories. We critically review and analyze conceptual material, along with clinical and research evidence relevant to the experiential and clinical realities of this population, to demonstrate the limitations of a biomedically based diagnostic–therapeutic paradigm at the expense of a developmental and disability-oriented perspective. Our main aim in this paper is to argue for an explicit recognition of both disease and disability perspectives, and a more balanced and appropriate deployment of these concepts across the continuum of clinical services, research, policy-making and professional and public education in relation to children with neurodisability; we also provide concrete recommendations to advance this progressive strategy. The relevance of these aims and strategies, however, extends beyond this particular population.

## Introduction

Many child-onset chronic health conditions are associated with functional limitations (traditionally referred to as “disabilities”). For some of these conditions, the presence, or, perhaps more accurately, the experience of disability, is a defining characteristic. The term neurodisability refers to “a group of congenital or acquired long-term conditions that are attributed to impairment of the brain and/or neuromuscular system that create functional limitations, including difficulties with movement, cognition, hearing and vision, communication, emotion, and behavior” (1). Historically, this population has been variably referred to as children with neurodevelopmental *disorders* or with neurodevelopmental *disabilities*, with these two terms often used interchangeably (2–6). Although changes in usage may arise following adoption of the term “neurodevelopmental disorders” as a specific category in the Diagnostic and Statistical Manual of Mental Disorders, 5th Edition (DSM 5) (7), in this essay, we argue that conceptual and empirical advances have made it more important than ever to differentiate the terms “disability” and “disorder.”

The long-standing, yet anomalous, practice of using two terms interchangeably to refer to the same population of children remains worthy of critical exploration regarding what it may reveal about the perceptions and attitudes that underlie both health-care practice and policy. One explanation is that disorders and disabilities are two terms that mean the same thing, and so are thought to be interchangeable (Explanation 1). Another is that they mean and connote different things, but widespread lack of awareness of the differences (as discussed in this essay) allows them to be conflated in general use (Explanation 2). The third possibility is that the people referred to, and specifically children as the focus of this paper, have (or are assumed to have) *both* disorders *and* disabilities, so either term is thought to be acceptable. In this scenario, it is possible that use of one or the other term may be more appropriate, but guidelines for this are lacking (Explanation 3).

This paper explores these possible explanations. It proposes that discrete perspectives about disease and disability underlie approaches to everyday clinical work, policy-making, and research with populations affected by chronic conditions, including children with neurodisability, but the distinctions between them may seem subtle and not worthy of critical scrutiny. We propose that these perspectives can and should be clearly identified and then explicitly applied to inform clinical practice and policy planning. The rationale for this proposed “parsing” of the language is addressed through four themes.

First, we elaborate on the argument concerning the distinctness of the *concepts* of disease and disability by describing their relationship within the World Health Organization’s (WHO) International Classification of Functioning, Disability and Health (ICF) (8), widely considered to be the contemporary reference conceptual framework for disability. We then outline the main characteristics and epistemic underpinnings of disease and disability *perspectives* in health care, as we see them. Second, we review the “goodness of fit” of each perspective in relation to childhood neurodisability, with reference to clinical realities of the field, emerging evidence, and trends in advocacy. Our third theme is a review of each perspective’s current use and place in relation to childhood neurodisability, along with some factors that contribute to the *status quo*. Finally, we conclude with suggestions for moving forward in order to achieve a more appropriate balance in deployment of each perspective; this includes practical strategies to promote explicit awareness, and appropriate adoption/implementation of disease and disability perspectives in child health and neurodisability.

At the outset, we need to point out that the perspectives on disease and disability discussed in this paper are not identical to the medical and social models of disability that have been thoroughly described elsewhere (9–11), although there is overlap with these approaches. The latter represent conceptual views or models of what disability *is*. The present paper takes ICF to be *the* contemporary working framework to conceptualize disability within health, and aims to demonstrate and elaborate on how distinct disease and disability perspectives subtly but importantly shape thinking and action on the part of health-care providers, and notably, physicians, as well as researchers, educators, and policy makers. Through a process that highlights and makes these perspectives explicit, it is hoped that we may actually update and go beyond previous positions on biomedical vs. biopsychosocial models in health care (12), thereby facilitating improvement in service delivery, policy making, and outcomes for persons with chronic health conditions associated with disability. As mentioned, our particularly focus is on children with neurodisability and their families.

## Theme 1: Disease and Disability Concepts and Perspectives

### Disease and Disability Terms and Concepts in the Era of ICF

Since its release in 2001, ICF has become the predominant conceptual framework for the human experience of health and disability (13, 14). While certain aspects of the ICF (such as the actual classification – the “C” in ICF) remain subject to critical commentary (13), the framework is increasingly used to organize scholarly as well as clinical activities. Key features of ICF that significantly advance the conceptualization of disability within health are that (i) disability is framed in functional terms, as the converse of healthy functioning; (ii) functioning is understood to manifest at the level of body, person, or person-in-society, operationalized, respectively, as intactness (or impairment) of “body structures or physiological functioning”; ability to carry out daily activities (“activities” or limitation thereof); and “participation” (or restrictions) in meaningful activities with other people; (iii) disability is not “in the person” but results from interactions between health conditions and contextual (usually environmental) factors; and (iv) functioning, health conditions (diagnosed diseases and disorders), and contextual factors (both environmental and personal) are each distinct and interconnected components of health.

Insofar as the ICF framework depicts diseases (or, more accurately, “health conditions”) and disability as distinct elements, the notion that disease and disability are the same and hence interchangeable (Explanation 1) is not viable in the ICF era. Illustrating this notion are the separate WHO international classification systems that exist for health conditions (diseases and disorders) and disability, namely, the International Classification of Diseases (ICD-10) and ICF, respectively. While intended by WHO to be taken as complementary (8), the distinctness and complementarity of diseases and disability have not always been so clearly set out. In fact, earlier attempts at classification of disability (15) tended to see disability as merely a “by-product” of diseases or disorders, reflecting perhaps the pre-eminence of a biomedically based diagnostic–therapeutic model in the twentieth century. The distinctness and complementarity of current ideas can be highlighted, and thereby better actualized, by dissecting out and making explicit the perspectives on disease and disability in health care and health services.

### Disease and Disability “Perspectives” – Characteristics and Underpinnings

The key components and characteristics of what this paper identifies as disease and disability perspectives are summarized in Table 1, along with a proposed designation of the epistemic underpinnings (or theories of knowledge) of each perspective.

**Table 1:** **Disease and disability perspectives: components and characteristics**.

	Disease perspective	Disability perspective
Focus, terminology, and taxonomies	Health conditions: diseases and disorders as seen through a biomedical lens	Disability (or disabilities): function and functioning as seen through a social impact lens
	“Diagnosis” arrived at by “ruling out” alternate explanations for the problem	“Formulation” arrived at by “ruling in” relevant positive and negative elements of a person’s situation
	Classified and cataloged in ICD-10 and DSM 5	Components and classification covered in ICF
Underlying conceptual basis and intervention paradigm	Biomedical (paradigm) diagnostics and therapeutics (“diagnose-and-treat”); treatment based on understanding of underlying biological derangements (pathophysiology). Hence, a strong reliance on biomedical science as the language of disease	Biopsychosocial model, which takes account of various factors within the person and their environment, in addition to biological derangements. Hence, the language of the social model of “person-in-environment”
Aims of intervention	Treatment aims to halt, and where possible reverse, underlying pathological processes; ultimately, if possible, to cure or at least control the condition	Rehabilitative interventions aim to mitigate effects of disease or disorder; to improve/optimize functioning though focus on the individual’s skills and environmental supports and adaptations; and to enable people to try to achieve their own life goals in their own ways
Proposed epistemic underpinning	Nomothetic	Idiographic
	Aims for knowledge that is broadly applicable through study of things/phenomena as *categories* (see text)	Aims for knowledge that helps to understand phenomena through detailed description of *individual* experience (see text)

Disease and disability perspectives clearly differ in their chief characteristics and appear to be underpinned by different epistemic foundations, which we believe to be represented best by the “nomothetic” and “idiographic” approaches to knowledge described by Austrian philosopher Wilhelm Windelband (1848–1915) (16). The *nomothetic* approach to knowledge refers to and emphasizes how knowledge can be generalized, extended to categories of things or phenomena, thus framing the knowledge generated in this way in terms of universal principles. This approach is best suited to the study of objective phenomena and hence finds most appropriate application in the natural sciences. *Idiographic* knowledge, on the other hand, is primarily focused on the individual case (hence “idio-”), whether a particular person or phenomenon. The emphasis is on the meaning of contingent, unique, and subjective phenomena relevant to the individual. This approach is therefore most closely identified with (and appropriate for) the humanities (16, 17).

The nomothetic approach to knowledge as described by Windelband seems to form a natural epistemic base for the disease perspective, with its focus on diseases as phenomena in the natural world, organized into categories in which each has its own etiology, pathophysiology, natural history, classification, and epidemiology. The disability perspective, on the other hand, is primarily concerned with variations in functioning among individuals, and the contingencies that underlie these variations. The aim is to describe how a person is functioning – at the levels of body, person, and person-in-society – and to account for an array of contributing factors (biological, personal, and environmental), the effects of which may be mitigated to optimize *that person’s* functioning. The disability perspective therefore fits closely with idiographic description and knowledge. And though an idiographic approach has been identified with the humanities, we can envision such as approach as one that aims for synergy between scientific and humanistic currents in health care (18–20). In the current era, some of the most effective and moving medical writing has been crafted by master clinicians with an eye and ear for the human dilemmas that make each expression of a “disease” unique and compelling (21–24).

## Theme 2: Disease and Disability Perspectives: Their Goodness of Fit within Childhood Neurodisability

### Goodness of Fit in Relation to Clinical Realities of Childhood Neurodisability

A biomedical diagnostic–therapeutic orientation to health care is integral to current versions of the disease perspective; its nomothetic basis is evident in the categorical way in which diagnoses and treatments are approached. Identification of a diagnosis leads directly to planning and implementation of “treatments” and “interventions” that wherever possible are meant to be specific for that category of disease or risk factor. This approach has been remarkably effective in management of many health conditions in clinical care (25), especially acute conditions, among which streptococcal pharyngitis may be considered prototypic. A patient’s symptoms (fever and sore throat) lead to diagnostic-specific identification of a condition (“Strep throat”) whose etiology and pathophysiology are well understood, allowing for targeted treatment (antibiotic with appropriate sensitivity to this strain of Strep) to halt and reverse the disease process and its effects in a fairly specific way. This approach has also been instrumental in public health control of diseases such as infections, nutritional deficiencies, and certain cancers, once the underlying causal pathways and biological derangements have been identified and found to be remediable or reversible (26, 27).

Childhood neurodisability, however, has two key characteristics that substantially reduce the “goodness of fit” of this approach. First, despite the plethora of labels in this field, the diagnoses among children with neurodisability convey limited knowledge of underlying biological processes toward which specific treatments might be directed with the intent of reversal or biomedical cure. Second, the mainstays of “treatment” are a range of (re)habilitative (development-promoting) and supportive services aimed at the person (and their family) and the environment, in order to promote adaptation and optimize functioning in ways that reflect that person’s interests and capacities. In the following paragraphs, we expand on what is distinctive about diagnoses and treatments/interventions in childhood neurodisability.

“Diagnoses” of children with neurodisability can be sorted into two main kinds: *developmental*, based primarily on describing functional characteristics within classical domains of child development, and including an alphabet soup of labels such as ASD, ADHD, ID, or DCD (representing autism spectrum disorder, attention-deficit/hyperactivity disorder, intellectual disability, and developmental coordination disorder, respectively); or *medical*, named on the basis of a primary underlying etiology – examples include Down syndrome, Fragile X, and fetal alcohol syndrome. The first kind of diagnoses have much in common, and indeed overlap, with mental health/psychiatric conditions, such as anxiety and depression. All of these are phenotypically and phenomenologically descriptive, “polythetic-categorical” entities (28). They identify constellations of features consisting of observable behaviors and/or subjectively reported feelings or phenomena. To be included in a developmental diagnostic category (disorder present – yes/no), a person must have a minimum number of defining characteristics out of a larger set, in which no individual feature is necessary or sufficient on its own. These diagnostic categories are organized in taxonomic systems such as DSM (7), but DSM was never intended by its authors to be informative as to etiology (28–30). Diagnoses of the medical–etiological kind, on the other hand, usually require presence of specific biomarkers and are often strongly informative as to etiology. However, they convey limited information about a diagnosed individual’s specific functional phenotype or the impact of that diagnosis on that person’s life. Most relevant to this discussion is that neither kind of diagnosis provides information that can be readily slotted into a biomedical diagnostic–therapeutic model. Advances in neuroscience have helped to identify broad trends and tendencies in brain structure and function among persons in diagnostic categories spanning the neurodevelopmental–mental health divide, such as ASD (31, 32), but this information is limited in its ability to predict malfunctioning at the biological or physiological level within individual children (33, 34). In addition, because many of these diagnoses identify conditions that are genetic or teratogenic in origin, given the current state of therapeutic technologies, a medical–etiological diagnosis often does not provide a guide to interventions aimed at halting or reversing specific pathological processes. An important exception is the small group of inborn errors of metabolism that may be amenable to specific intervention capable of halting and possibly improving disability (35). In the future, technological advances in “precision medicine” may allow knowledge of specific genetic etiology to be used to tailor biological treatment approaches for certain conditions, as is happening already in epilepsy (36). At present, however, knowing a diagnostic category rarely leads to specific therapy along the lines of the streptococcal pharyngitis example used earlier.

Another feature of neurodevelopmental disorder/disability diagnostic categories, whether developmental–functional or medical–etiological, is the stunning diversity and heterogeneity in clinical characteristics among children who fall within a category and the clinical and functional overlaps between categories. This is in part a function of categorical-polythetic diagnoses in which symptom profiles can vary markedly across individuals who all meet criteria for membership of a diagnostic category (29, 34, 37, 38). This reality is reflected, for example, in increasing use of the term “spectrum disorders” to describe neurodevelopmental conditions; references to “the autisms” as a more authentic term than the more traditional “disease”-based designations (39); and the emphasis in contemporary definitions of cerebral palsy (CP) as a “heterogeneous group of disorders” (40). Adding to this source of diversity and heterogeneity among diagnosed children are the twin phenomena of “comorbidity” and “functional complexity” now recognized as another hallmark feature of children with neurodisability (41–46).

The consequence of all of these considerations is that, in the context of childhood neurodisability, a diagnosis may tell us relatively little about the individual person and their abilities, let alone their needs. As a result, in contrast to the role that diagnosis plays in the biomedical diagnosis–therapeutic paradigm that currently characterizes the disease perspective, a diagnosis of ASD, ID, or 22q11 deletion syndrome cannot be assumed to lead naturally to the design of interventions that are specific to, appropriate for, or capable of meeting the needs of the diagnosed child diagnosed, or his or her family. Indeed, commonly used phrases such as “treatment of ASD” may serve to perpetuate a misconception, even a myth, in child health. In the real world, we usually have to help families “manage” a child with a diagnosis of ASD, or support their different development. That child may differ substantially from another child diagnosed with ASD in terms of primary symptomatology and in the presence of absence of additional diagnoses, or perhaps just features, of ADHD or ID. Furthermore, the child’s family may experience considerable psychosocial stress, which, if not addressed, will make it difficult to create an environment supportive of positive development change.

This is not to say that biomedical diagnosis is unimportant within childhood neurodisability. Determining that a child has ASD, ADHD, or 22q deletion syndrome can be extremely important for a host of needs/purposes ranging from direct treatment in cases of inborn errors of metabolism to prevention through genetic counseling and to facilitating communication with parents and among professionals and affected persons. Diagnoses are also critical for administrative and epidemiological purposes and for addressing the important human need to “put a name on it” (47). Naming a condition in turn allows people to identify themselves and deal with their predicament adaptively through learning about it and connecting with others. However, it cannot and should not be assumed that a descriptive diagnosis of ASD or ADHD, or even a situation in which a specific etiology is known or suspected for a functionally defined condition (such as ID due to Down syndrome, or which occurs as a complication of neonatal adversity such as extreme prematurity), leads naturally to the design of interventions that are specific to, appropriate for, or capable of meeting the needs of an individual person diagnosed with ASD, ID, or 22q11 deletion syndrome.

Turning to the nature of treatments and interventions for children with neurodisability, these mainly reflect the habilitative (developmental) focus that is integral to the functional/disability perspective, coexisting with treatments that target known and understood pathophysiological (biomedical) mechanisms. One major class of interventions includes educational and behavioral strategies, and another comprises medications, surgeries, and specialized equipment and surgeries. But whatever the class, it is rare that treatments and interventions target an underlying etiopathological (biological) process that is specific for diagnosed disorders, such as ASD, ADHD, and DCD, language disorders, CP, Fragile X syndrome, or fetal alcohol spectrum disorder (FASD). Psychologically based interventions that aim at teaching skills or changing behavior are widely used in childhood neurodisability, especially for the less visible, less physical challenges. Within these broad categories are more specific therapy types, such as applied behavioral analysis (ABA) under the category of behavioral interventions, the Orton–Gillingham approach under educational interventions, and cognitive behavioral therapy (CBT) under psychological interventions. All of these are supported by evidence of effectiveness, but none of which is truly specific to any one disorder. ABA is most-widely known for its effectiveness when used with children diagnosed with ASD (48), but ABA, and the associated early intensive behavioral intervention (EIBI) approach, has been found to be effective in other situations too (49). More broadly, psychosocial and family-based approaches are known to be effective in management of many childhood disorders (50–52). In terms of educational interventions, strategies such as providing a student with visual supports is recommended and helpful for any child whose learning is affected by auditory or language weaknesses, regardless of any particular medical diagnosis. Mental health conditions occur far more frequently among children and youth with ID and neurodevelopmental conditions than among typically developing children (53), and CBT has been found to be across a wide range of mental health disorders in children and youth (54). In other words, the best of these many interventions are not condition-specific; rather, they address symptoms and behaviors.

Medications, often seen as the prototypical “biological” intervention, rarely have the kind of specificity that is such a hallmark of the biomedical diagnostic–therapeutic strategy (55) typified by antibiotics for streptococcal pharyngitis or insulin for type I diabetes, when used in the course of management of children with neurodisability. Stimulants such as methylphenidate, a commonly used treatment for ADHD, may also be efficacious in neurological dysfunction post-traumatic brain injury (56); baclofen, most closely associated with CP, may be useful for management of spasticity regardless of cause (57), while clonidine, often used adjunctively in ADHD, may also be useful for spasticity following brain injury (58). Similarly, psychopharmacological agents including the so-called antipsychotic drugs are used in management of children with various neurodevelopmental conditions to help control aggressive behavior, agitation, or irritability (59, 60), just as the SSRI “antidepressants” are useful in management of anxiety and/or compulsive and repetitive symptomatology (61) in the absence of psychosis or depression. At the present time, treatments that successfully affect CNS functioning, whether biologically active or experience-based, do so in ways that cannot always be predicted based on a specified diagnosis. Conversely, when prescribing an SSRI on the basis of experiential evidence of effectiveness, the prescriber does not know whether serotonin is lacking in the specific patient’s brain pathways.

More broadly, these comments fit with a rising tide of evidence for the benefit of “lifestyle” interventions for various developmental, behavioral, and mental health conditions. For example, exercise and physical activity have been shown to be effective as part of a set of interventions for ADHD (62) but not limited to ADHD; efficacy has been demonstrated also for depression (63) and will likely be shown for conditions too. So while it is fair to say that exercise or physical activity has a therapeutic effect in ADHD or depression, a view that better fits all the available evidence is that exercise and activity appear to have salutary effects on brain functioning in general and perhaps even more clearly in certain situations of disrupted or dysregulated brain function. We can conclude by saying that evidence for the effectiveness of many interventions for children with childhood neurodisability and many mental health conditions is usually derived from condition-based studies and so is presented as a treatment for condition A or B; in reality, however, the interventions themselves are far from condition-specific.

### Goodness of Fit in Relation to Emerging Evidence and Contemporary Advocacy in Childhood Neurodisability

A body of empirical evidence is emerging to support the notion that, in relation to neurodisability in children and youth, a disease perspective that places a person’s diagnosis at the center is less relevant (or appropriate) for planning interventions, services, and supports than a disability perspective in which individual functional characteristics are carefully considered as a starting point for “management.” Recent evidence builds on seminal findings of Stein and Jessop that for children with chronic health conditions, diagnostic category may contribute little to our understanding of the impact, needs, or psychosocial correlates of the condition in that individual (64). Subsequent work includes the illustration that, in a large population of children and youth with CP, neither the topography nor the type of motor impairment of the CP predicted their functional abilities (65), as described with a valid and reliable functional classification system (66). More recent demonstrations of this idea include studies that contrast the role of functional characteristics against established diagnosis in predicting need and use of supports and services, and conclude a centrality/primacy for functional characteristics (67–71). Finally, evidence from clinical- and population-based studies shows that child functional characteristics are more informative than child diagnosis status in explaining various child and family health and health-related outcomes among children with ASD and other NDD (72, 73).

Account also needs to be taken of contemporary stakeholder advocacy movements that strongly emphasize – when ascertaining need for services and supports among persons with impairments – the importance of adopting a functional approach that fits with our notion of a disability perspective. This is perhaps most clearly articulated in the approach advocated by American Association on Intellectual and Developmental Disabilities (AAIDD), using tools, such as the Supports Intensity Scale (74, 75), that clearly are idiographically informed. Though more adult oriented, this approach has increasing relevance and is being investigated psychometrically among children and youth with disabilities (76).

## Theme 3: Disease and Disability Perspectives: Current Status within Childhood Neurodisability

Theme 2 demonstrated that basic clinical realities of childhood disability, along with an emerging evidence base and increasing advocacy efforts, converge to make the traditional biomedical diagnostic–therapeutic approach, which is so central to the “disease” perspective, less appropriate for the context of childhood neurodisability than an ideographically informed disability perspective. In this section, we briefly examine the current status of health-care practices, policy making, and research in relation to that conclusion and review key factors that we consider to contribute to maintaining the *status quo*.

Conflation of disease and disability perspectives, and the imbalance between them, continues to be prevalent in health care and services planning. Conflation is most obvious in commonly used terminology. Beyond the example of the interchangeable use of neurodevelopmental disorders and disabilities that served as an important case-in-point and gateway into this paper, it is commonplace to hear that children with autism or Down syndrome “have – or even suffer from – a disability.” According to ICF, however, autism and Down syndrome are “health conditions” that the child “has,” while disability (or disabilities) is more precisely a state a person experiences and which manifests as impairment in aspects of body structure, or function, or in activity limitations or participation restrictions. For example, the child with Down syndrome may experience cognitive impairment, and the child with ASD may have social and/or adaptive impairment. But this is different from the implication that DS and ASD are, in themselves, disabilities. Conflation in common usage reflects, but also perpetuates, lack of precision and distinctness among these conceptually different perspectives. The fact that some terms actually straddle the disorder–disability divide further obscures these nuances. For example, “intellectual disability,” which primarily describes challenges in the functional areas of cognition and adaptive skills, and so is perhaps more accurately termed cognitive–adaptive (or intellectual) impairment (77), is itself codable as a “health condition” in DSM-5 under the hybrid rubric of ID (intellectual developmental disorder) (7). To go back to the explanations offered earlier in this paper, conflation maps on to Explanation 2: there may be some awareness of a difference between disease and disability perspectives, but sensitivity to the difference outlined in ICF, at least in terms of the language used, is lacking. The effect of this conflation is to reinforce, powerfully but insidiously, the primacy of disease perspectives (78), and to obscure the relevance of disability perspectives with their functional focus and social and implications for management and adaptation.

Imbalance between disease and disability perspectives is also seen in situations where a child’s diagnostic status may be used as the main or only determinant in planning for and regulating access to “ancillary and enabling” services and supports (79), with scant attention to the individual child and family’s functional characteristics and needs. Examples of this are found across jurisdictions and in relation to rehabilitative, supportive, and special educational services, such as British Columbia’s specialized funding programs for children with special needs, which include two specific conditions, ASD and FASD (80); diagnosis-based eligibility criteria for early intervention services in Australia (81); and the targeting of specific neurodevelopmental diagnoses in Swedish legislation that regulates access to services (82). This situation is regrettable but also ironic in light of the increasing emphasis on both patient-centered care (83). Further examples from outside the health-care realm are the use of medical and other kinds of categories in classifications that determine children’s eligibility for special educational supports (84, 85). These “other categories” are often referred to as disability categories, but as the preceding discussion will have made clear, they are more accurately framed as impairment categories.

Imbalance is also seen in clinical situations, when physicians may devote almost all of their energy toward attempts to identify a medical etiology for a child’s difficulties, and very little toward a more holistic formulation of functional strengths, limitations, and child and family needs. Sometimes, this professional activity extends to seeking and applying politically “useful” diagnoses to enable families to access services apparently reserved for people with that specific diagnosis (86); further consideration of this vexing situation goes beyond the scope of this paper, however.

In parallel, we see similar imbalance in the research field, where much of the funding for neurodevelopmental disorders/disabilities goes to selected conditions such as ASD, with much of that research directed toward elucidating the underlying neurobiology of the condition (34, 38) and therefore expressing a disease (or disorder)-based perspective. It is possible that as precise mechanisms become elucidated, and if these mechanisms prove to be amenable to specific treatments, management of children with neurodisability will come to comprise a larger component of biological treatment aimed at reversing specific underlying pathological processes. However, we are reminded that an exclusive focus on the biomedical nature of a neurodevelopmental condition, such as ASD, has not solved the daily living challenges of affected persons (38). Indeed, short of completely eradicating the underlying chronic condition, this approach is not likely to do so without individualizing the broader “management” of the conditions, in common with other chronic “medical” conditions such as diabetes. Even when the focus of research is not on underlying biological or biomedical processes, but rather on effects and correlates of developmental and mental health conditions, such as impact on child and family which have been shown to have important commonalities across conditions (64), research outputs still tend to reflect the disease or condition-specific silos from which they arise (87, 88).

A number of factors underlie and perpetuate the conflation and imbalance which characterize the *status quo*, of which we consider three briefly here. The first is simply a reflection of the relatively recent arrival of ICF into health care with its expanded range of ideas. The biomedical diagnostic–therapeutic approach has become established over a century and a half as the dominant paradigm in medical practice, education, and training (89), whereas the ICF framework was unveiled only 15 years ago. Traditional models of medical education may continue to emphasize a biomedical diagnose-and-treat approach, and the resources to formally support learning about disability and rehabilitation often remains lacking (90). Innovative ideas and concepts tend to diffuse out, be debated, and taken up slowly into practice and policy, especially when the current state of health care, education, and policy reflects a culture that is oriented toward, and perpetuates, a nomothetic disease perspective over an ideographic disability perspective.

This leads into the second factor, which is the existence of structural constraints in publicly funded service systems. In Canada, for example, the national health insurance program, known as Medicare (Canada), is designed to fund medical services and treatments, primarily physician or hospital-based services and interventions such as medication and surgery. This federal legislation renders as out of scope the “ancillary and enabling” services needed by persons with disability (79). In Canada, this gap is filled by provincial jurisdictions in a variable and piecemeal way across the country. The legislative-structural constraints described for Canada likely have analogies in other parts of the world. Their existence speaks to the very definition of health that law makers and societies use in formulating policies. When *health* policy remains narrowly defined, it will be less relevant to children with neurodisability and to adults and children who live with chronic conditions associated with disability. In the final theme of this paper, we propose thinking about “health and social policy” when it comes to persons living with disability.

Finally, there is the contribution made by administrative factors. Identifying health problems using diagnostic codes is a relatively simple and convenient way to summarize, capture, and store clinical information as required for administrative purposes, such as utilization and billing for medical services, quality improvement, and epidemiological studies. One criticism that has been leveled at the disability perspective, and ICF in particular, is that it does not come with an easily implementable way of dealing with administrative needs in terms of convenience, efficiency, and succinctness (13). On the other hand, there has been increasing interest in development of functional classification measures with potential to fill this gap; we return to the topic of data collection and ways to address the other factors outlined here that maintain the *status quo*, under Theme 4.

Although in this section we have focused on conflation and imbalance as characterizing the *status quo*, it is not infrequent to find broader and more “progressive” deployment of disease and disability perspectives in policy, delivery, and education in health care. One example is evidence of increasing uptake of ICF domains in more recent population-based disability surveys, as reported from Ireland (91). In many jurisdictions, too, a “blended” approach exists for determining eligibility for services and supports for children with neurodisability and their families, resulting in a mix of policy approaches from diagnosis-specific to those emphasizing functional characteristics. Foundational pieces of legislation in the United States illustrate this. The Individuals with Disabilities Education Act (IDEA) adopts a largely categorical approach to children’s service eligibility, except for children under 3 years of age who are eligible by virtue of having, or being at risk for, developmental delay which is not further specified. Alongside this blend of approaches in IDEA is the Americans with Disabilities Act (ADA), in which disabilities are broadly (and non-categorically defined) as significant physical, intellectual, emotional, or health limitations that interfere with life activities (Turnbull, personal communication). In Canada, a recent review of criteria for an array of (re)habilitative and special educational services and supports for children across the country found that early intervention services are generally functionally based, while access to behavioral interventions are largely diagnosis-based, certainly for preschool age, but also for school-aged children. ASD and, in some provinces of Canada, FASD, are the main diagnoses that “open the door” (92). For school-aged children, it is most common to find a combination of diagnosis and function-based approaches used such that diagnostic categories are used to confer funding for supports and services to school districts, with these supports then delivered to individual students based on their functional needs (92).

## Theme 4: Moving Forward: A Progressive Agenda for Deploying Disease and Disability Perspectives in Child Health and Childhood Neurodisability

### Can We Articulate a Clearer Balance between Disease and Disability Perspectives?

Before we embark on recommendations to shift the *status quo* on perspectives and approaches to persons with chronic health conditions associated with disability, we return to a point made at the beginning of this paper. Explanation 3 for the interchangeable use of the terms neurodevelopmental disorders and neurodevelopmental disabilities was that both terms may (at times) apply, but it is not clear when to use one and when to use the other. This explanation fits with the way the ICF framework depicts health conditions coexisting and dynamically interacting with functional aspects of health (or disability), and with contextual factors, even though the presence and fact of a chronic health condition does not necessarily mean a person experiences “disability.” Throughout this paper, we have argued for a greater emphasis on a “disability” perspective, in both its functional and contextual dimensions, assessing the day-to-day *implications* of a health condition for child and family. This includes aspects of health and social policy relevant to regulating access to services and supports. While this next point is perhaps obvious, we reiterate here the importance of a disease perspective in (i) clinical situations where, in the diagnostic phase, the aim is to identify an underlying medical diagnosis, with potential genetic, epidemiologic, and prognostic implications and (ii) in research efforts that aim to understand the biological determinants of a patient’s predicament. Having a diagnosis can have important implications for treatment and prevention, as well as construction of social identity and psychological “closure” (47), as has been noted earlier, while knowledge of biological mechanisms advances the possibilities of prevention and cure.

### Practical Steps/Strategies to Promote Explicit Awareness and Appropriate Implementation of Disease and Disability Perspectives in Child Health and Neurodisability

Shifting the *status quo* on perspectives and approaches to persons with chronic health conditions associated with disability is an important endeavor. It emerges as a natural consequence of all that we have argued thus far about imbalance and neglect of the disability perspective in current practice, policy, education, and research. It is made even more compelling by an increasing body of evidence that demonstrates how, for children and adults affected by chronic health conditions, the presence of disability demarcates differences in impact and needs in a very robust way (93–96). The task ahead is to disentangle conflation, to recognize the complementarity of the disease and disability perspectives in the context of childhood neurodisability (as well as child health, and human health, more generally), and to see how each contributes to a deeper understanding of both individuals with impairments and to the field in which we work. We have argued that the first step in this long journey is to bring attention to, and highlight the *notion* of disease and disability perspectives, and how they underlie and shape much of what we do in the clinic, the laboratory, and in policy making, even if under-acknowledged (when not ignored completely). The next step is to ensure that both perspectives are actively considered at all times, with due regard to the question of when one perspective might claim primacy of place over the other.

The final part of this paper sets out practical steps – including actions/changes across a number of fronts – that we believe are necessary to achieve a new balance in activities that concern children with neurodisability and their families. We organize these ideas, suggestions, and strategies under the headings of clinical practice; data collection approaches; tools to capture data on both health conditions (diagnosis) *and* function; health professional education; organization and prioritization of research funding and programing; and policy approaches.

#### Clinical Practice

Several changes can be instrumental in moving forward with recalibrating the weighting we give to disease and disability perspectives in clinical practice. At the level of basic organizational models of service, we recommend a shift toward non-categorically defined clinics for children with neurodisability, based on functional rather than diagnostic considerations. In this scenario, a clinic’s mandate may be, for example, to assess and advise families of children with communication impairments, rather than children with a particular diagnosis, such as ASD. Condition-specific clinics, such as those for autism or CP, are popular but tend to arise in response to the passions and pressures of interest groups, both professional and public/consumer. Though such clinics may be endowed with a significant body of expertise in relation to that condition, which may be necessary in some circumstances (e.g., a specialized multidisciplinary clinic dealing with children with spinal cord damage), for prevalent neurodevelopmental disorders, the clinical realities of heterogeneity and complexity pointed out in Theme 2 make such a model less appropriate. They may also disenfranchise, and make “orphans” of, children who need clinical services to address phenotypically similar challenges but who do not appear to fit with the clinic’s condition-specific mandate. On the other hand, “non-categorical clinics” for children with what we may call complex neurodevelopmental conditions would have a pool of the expertise needed to deal with the challenging behaviors, learning issues, and family impacts that occur in common across the full range of conditions. This would happen irrespective of the specific genetic or teratogenic etiology and would be open to children irrespective of these considerations. Movement in this “non-categorical” direction is seen in the contemporary “complex care” movement, particularly strong in the United States and Canada, with a tremendous upsurge of interest in organizing clinical services based on a clinical population defined by complex service needs rather than by a specific diagnosis, or the multiple diagnoses, that affected children may have (97). Part of making a non-categorical approach successful will be educating parents, professionals, program managers, and policy makers about its rationale and value, and the need to balance diagnostic with functional considerations without “taking away” a diagnosis for their child.

The next step is to ensure that, within clinics that assess children with possible neurodisability, *the diagnostic phase* involves processes designed to make both a biomedical diagnosis (developmental and/or medical–etiological) and a clear functional assessment that describes and summarizes – essentially formulates – the most relevant functional information. How we might move toward achieving this is covered below and will clearly involve implementation of the ICF concepts in clinical work, along with the consistent use of relevant functional assessment tools. As current examples of “work in progress” one of us (Peter Rosenbaum), in collaboration with PhD students, is presently working on developing and evaluating modules for parents of children with CP to introduce these concepts to them (98), and also on developing an “F-words” hub, along with parents members of the research team, to make ideas about the “F-words in childhood disability” (99) easily accessible and useable. Parents, of course, represent one force by which the system can be changed from the “bottom up.”

In its turn, *the treatment/intervention phase* must explicitly cover all aspects of intervention and supports, including medical or surgical treatments, specific therapies, equipment, and technologies, all designed to promote and optimize performance of daily activities and social participation, as well as measures to make the environment (family, school, neighborhood) as promotive for these things as possible. Below we refer to the F-words ideas that discuss function, family, fun, friendships, fitness, and future – concepts that can inform all our work with children with impairments and their families. Sometimes families perceive that, at least in the context of specialized hospital services for children with chronic conditions associated with disability, the ancillary and enabling services are neglected in a way that is very problematic for the family (100).

#### Data Collection Approaches and Tools

Part of implementing a disability perspective more fully and explicitly into clinical care involves paying more attention to functional aspects of health. There are various ways to achieve this. First is the need to incorporate ICF-based fields routinely in every clinical assessment for children with neurodisability. We can also consider the creation of an ICF-based template with which to identify an individual’s issues, strengths, and needs. An example of this has been created by the second author and his colleagues, building a set of “F-words” (function, family, fitness, fun, friends, and future) onto the ICF framework (99). The approach is strengths-based, family-friendly, comprehensive, and can be individualized by the person with the impairment, their family, and their clinical team. Such an approach has the benefit of reminding all the players to see these aspects of people’s lives and not simply the ICD-10 codes as a shortcut to the person.

Beyond the ICF, in the field of childhood disability, there are now several purpose-built valid and reliable functional classification systems, many in use around the world, which provide perspectives complementary to the diagnostic terms. Tools, such as Gross Motor Function Classification System (GMFCS), Manual Ability Classification System (MACS), Communication Function Classification System (CFCS), and Autism Classification System of Functioning: Social Communication (ACSF:SC), capture functional information (“profiles”) in a practical, convenient, and psychometrically sound way that may make them more suitable for the task than ICF as it is currently formulated (certainly with respect to the limited capacity of the ICF to “classify” functioning). The goal of creating a profile of the current functioning for each child will involve agreeing on a set of measures we should use for every person with a neurodevelopmental disorder/disability, without assuming that a child with CP simply gets the GMFCS and a child with ASD gets the communication scale ACSF:SC. Functional profiles can and should include a focus on “can-do” areas and strengths, guided perhaps by the F-words model/template (99). In this way, assessment will lead naturally to address the broad question of: What is “treatment” or “intervention” meant to accomplish? Whose goals are important? What will the child or youth be able to do – or do better – now than before? The questions asked will inform the choice of the tools to find answers. Other contemporary measurement tools designed to capture functional information, either in relation to ICF domains (101) or in terms of needed functional supports (74, 75), are presently available primarily for adults but are likely to prove useful as part of implementation of the disability perspective in child health with further validation in this population (76).

Ultimately, we believe that a matrix of diagnostic and functional descriptions – based on disease and disability perspectives, and on the intended purposes of the data generated – will enable clinicians, researchers, administrators, and policy makers to recognize explicitly the interconnections between these ways of talking about the children and youth to whom we are all committed. They will then be able to use this cross-linked information for their many purposes without confusing one perspective with another.

#### Professional Education

As we noted regarding clinic work, there are also several kinds of changes that can be instrumental in moving forward in the area of education to recalibrate how disease and disability perspectives are aligned and weighted. The following recommendations apply in the broadest sense to education not only of trainees in the health professions but also of the public at large and to policy and decision makers. These recommendations are particularly salient in medical education, which remains largely in thrall to biomedical diagnostic–therapeutics (89, 102), and in recognition of the important role that physicians and other health professional leaders play in influencing practices, perceptions of health, and policies. At a fundamental level, and regardless of the biomedical condition, we encourage discourse in learning settings to be framed in the language of “people with health conditions,” with a move away from a more traditional focus on teaching about diseases. Our next recommendation is that educators disseminate the *notion* of disease and perspectives (at a conceptual level), as well as teach and discuss the non-categorical approach in child health, including its strengths and limitations. We recommend that educators promote a wide awareness and use of ICF framework – at very least, in its conceptual side, e.g., teaching the ICF framework to health-care trainees (103). In a promising development, at McMaster University in Canada, a biennial academic course on the ICF is now offered at the School of Rehabilitation Science, and ICF has been recently introduced to the Faculty of Health Science’s Program in Interprofessional Education (104). Finally, clinician-educators can serve as role models for an explicit embrace of disease and disability perspectives by organizing our own clinical work and research with this dual perspective always visible, and through a willingness to let go of disease-based silos.

#### Organization and Prioritization of Research Funding and Programing

A main strategy here is to create a better balance between research done in diagnosis-based silos and research based on non-categorical approaches. Successes and frustrations of the non-categorical approach are discussed in a review of the 25-year history of the Research Consortium on Children with Chronic Conditions (105). Tangible progress in the direction of better balance can be seen in an increasing body of research, especially from the United States on “children with special health care needs” (106, 107), and the recent emergence of Complex Care as a focus of clinical and academic activities (97, 108). Research involving children with neurodevelopmental disorders/disabilities, or “neurodisability,” as a non-categorical endeavor, remains limited however, with some notable recent exceptions (1, 43, 109). Most child health research continues to be funded and carried out at disease or condition level. We have argued that core “clinical realities” of childhood neurodisability, including clinical heterogeneity and comorbidity/complexity, detract from the validity and meaningfulness of work organized in this way. Even studies that may best be construed as advancing knowledge about children with neurodisability broadly, in which findings really speak to the effects of children’s functional characteristics in relation to consequences and implications, are often reported in a way that emphasizes the significance of one condition over another, a situation we have discussed elsewhere (72). In this essay, we have referred to the strong evidence of the commonalities across biomedically distinct chronic conditions. While in no way calling for an end to biomedical research into these myriad conditions, we believe that there are important opportunities to aggregate parental and child experiences across these many conditions as a basis for increasing the numbers of people contributing to the research endeavors and for capitalizing on the application of the findings. These efforts will also serve to demonstrate to clinicians, families, service providers, and policy makers that the traditional balkanization of service and research activities is limiting and out of date with modern thinking and should be abandoned.

#### Policy Approaches

As has been noted elsewhere in this paper, in the area of policy making in chronic childhood conditions, we recommend replacing policies regulating access to services and supports based on diagnostic category with a less categorical and more disability-based perspective-oriented approach. Such an approach places *functioning* (at level of body, person, and person-in-society) and the person’s *context* as the central considerations. The first step toward this move will require conversations with policy and decision makers to explain the rationale based on the arguments made in this paper, including the notion of “disease and disability perspectives.” This is the responsibility of clinicians, educators, and leaders in health services research, who will also need the skills to anticipate “real world” political pressures that decision and policy makers confront from health condition-related activism. This process can align itself, when appropriate, with ongoing lobbying/advocacy on the part of groups, such as American Association for Intellectual and Developmental Disabilities, for increasing use of needs-based assessments that look at functional limitations, such as SIS (74–76, 110), but also with due consideration given to functional strengths, and not only within the child but also the family and, ultimately, the community.

## Concluding Comments

A confluence of factors spanning evolution in core concepts, shifting priorities, and innovations in measurement make this a moment of historic opportunity, in our view, for decisive progress in health care and services not only for children with chronic health conditions typically associated with disability but also for persons of all ages. We hope our framing of diseases and disability perspectives may contribute to ongoing and productive discussion and progress.

## Author Contributions

AM conceived the paper and produced the first draft. Both authors contributed to writing and shaping the subsequent versions.

## Conflict of Interest Statement

The authors declare that the research was conducted in the absence of any commercial or financial relationships that could be construed as a potential conflict of interest.
